# Avian influenza outbreak in Turkey through health personnel's views: a qualitative study

**DOI:** 10.1186/1471-2458-7-330

**Published:** 2007-11-15

**Authors:** Ozlem Sarikaya, Tugrul Erbaydar

**Affiliations:** 1 Department of Medical Education School of Medicine University of Marmara, Istanbul Turkey; 2 Department of Public Health School of Medicine University of Yuzuncu Yil, Van Turkey

## Abstract

**Background:**

Avian influenza threatens public health worldwide because it is usually associated with severe illness and, consequently, a higher risk of death. During the first months of 2006, Turkey experienced its first human avian influenza epidemic. A total of 21 human cases were identified, 12 of which were confirmed by the National Institute for Medical Research. Nine of the cases, including the four fatal ones, were from the Dogubeyazit-Van region. This study aims to evaluate the efforts at the avian influenza outbreak control in the Van-Dogubeyazit region in 2006 through the experiences of health personnel.

**Methods:**

We conducted in-depth interviews with seventeen key informants who took active roles during the avian influenza outbreak in East Turkey during the first months of 2006. We gathered information about the initial responses, the progress and management of the outbreak control, and the reactions of the health professionals and the public. The findings of the study are reported according to the topics that appeared through thematic analysis of the interview transcripts.

**Results:**

Following the first suspected avian influenza cases, a Van Crisis Coordination Committee was formed as the coordinating and decision-making body and played an important role in the appropriate timing of decisions. The health and agriculture services could not be well coordinated owing to the lack of integrated planning in preparation for outbreak and of integrated surveillance programs. Traditional poultry practice together with the low socio-economic status of the people and the lack of health care access in the region seemed to be a major risk for animal to animal and animal to human transmission. The strengths and weaknesses of the present health system – primary health care services, national surveillance and notification systems, human resource and management – affected the inter organizational coordination during the outbreak. Open communication between the government and the public played an important part in overcoming difficulties.

**Conclusion:**

Although there were problems during the avian influenza outbreak in Turkey, the rapid responses of the central and regional health authorities and the performance of the health workers were the key points in controlling the epidemic. The lessons from this outbreak should provide an opportunity for integrating the preparation plans of the health and agricultural organizations, and for revising the surveillance system and enhancing the role of the primary health care services in controlling epidemic disease. Developing successful strategies based on knowledge and experience may play a valuable role in delaying an avian influenza pandemic.

## Background

The highly pathogenic Avian Influenza A (H5N1) virus has caused more than ten outbreaks worldwide and many related human fatalities have occurred since 1997. A total of 258 laboratory-confirmed cases have been reported in South East Asia, North Africa and Europe by the World Health Organization (WHO) and 154 people with confirmed avian influenza died between 2003 and 2006 [1,2]. Most patients have similar features; they are children, they have a history of close contact with poultry or wild birds, and they live in some of the poorest areas of the world. The human cases in Turkey and Azerbaijan were the first confirmed reports of human avian influenza infection outside Asia and Africa. This was alarming not only for Turkey but also for the European Union and other countries. After considering the epidemiological and laboratory evidence, WHO has maintained its pandemic alert at Phase 3 (of 6), indicating that the new influenza strain causes human infections with no or very limited human to human transmission [3].

Effective surveillance, early warning systems and containment measures based upon a general capacity for health care have been recognized by WHO as the essential action strategies [4,5]. In May 2002, "The Global Agenda for Influenza", which was issued by WHO, stressed the necessity of expanding animal influenza surveillance, and noted that studies at the domestic-wild bird and human-domestic bird interfaces are part of this activity [6].

The H5N1 type of avian influenza virus outbreak was lived in Turkey on January 2006; a total of 21 human cases were identified [3], 12 of which were confirmed by the National Institute for Medical Research [3,7]. Nine of the cases, including the 4 fatal ones, were from the Dogubeyazit-Van region. The confirmed cases were people aged 3–16 years who had close contact with ill poultry in the rural area in Dogubeyazit or in migrant wards in Van [3,7,8]. The timeline of events was summarized in Table 1.

**Table 1:** Timeline of the avian influenza outbreak in Turkey 2006

Dates	Events
8–26 October 2005	• Turkish Ministiry of Agriculture (MoA) announced the presence of H5N1 influenza in poultry in the north-western part of the country on 8 October ^a, b^. • On 13 October, World Organization for Animal Health (OIE) confirmed the presence of H5N1 in samples taken from patient poultry in Turkey^a^. • Deaths of chickens occurred in Dogubeyazit near the end of 2005, and a confirmed outbreak of H5N1 avian influenza in chickens and ducks was reported on 26 December^b^. • The Turkish Ministiry of Health (MoH) published the country's first national pandemic influenza action plan, developed on the basis of the WHO checklist for influenza pandemic preparedness planning^a^.
24–25 December 2005	• Three children in one family in Dogubeyazit, in Agri province, applied to the local outpatient clinic and treated for fever, sore throat, arthralgia and myalgia^a^.
30–31 December 2005	• The children's condition had worsened and was brought to Dogubeyazit State Hospital's by their father^a^. • The doctor of emergency room and consultant pediatrician in DSH accepted them as suspected avian influenza cases and transferred to the Van Yuzuncu Yil Univesity (YYU) Hospital^a, b^.
1–5 January 2006	• Samples taken from cases included in the ongoing influenza surveillance in the region were sent to the Turkish MoH, Ankara Hifzisihha Laboratories^a^. On 4 January the MoH received confirmation of H5N1 from National Influenza Centre at the Refik Saydam Hygiene Centre in Ankara and Capa Hospital in Istanbul^a^. • Turkish MoH notified the WHO Regional Office for Europe of the outbreak through the WHO Country Office^a^. • The Turkish MoH put its avian influenza preparedness plan in action^a, b^. • A regional meeting was held at the Van PHD to which the Governors of eight cities, the responsible Deputy Director of the PHD, communicable disease unit directors, provincial agriculture and rural affairs directors and animal health unit directors were invited. • Turkish and WHO experts assembled to review the epidemiological situation and provide further laboratory and clinical expertise^a^.
6–7 January 2006	• The National Institute for Medical Research which is the influenza reference laboratory and WHO collaborating center confirmed the presence of H5N1 in samples from the patients at YYU Hospital^a^. • The surveillance started in the region and the health team was assigned with the avian influenza surveillance from Ankara to Van and Dogubeyazit^b^. • The members of Turkish MoH Avian Influenza Scientific Committee interviewed with the experts of WHO, CDC and European Union^b^.
8–12 January 2006	• Turkish MoH and MoA teams moved to Van and organized the meeting with the authorities from nine provinces^b^. • Total 12 cases from Dogubeyazit (8), Van (1), Sanliurfa (1), Kastamonu (1) and Eskisehir (1) were confirmed by National Institute for Medical Research^b^. • Eight of confirmed cases were treated at Van YYU Hospital and the others treated at Diyarbakir Dicle University Hospital, Sanliurfa Children Hospital, and Agri State Hospital.

^a ^WHO [3]

^b ^Buzgan T [8]

^c ^Oner AF et al [7]

In this period, more than one hundred suspected human cases (21% of total suspected patient in YYU Hospital) underwent prophylactic therapy in the outpatient clinic and twenty five percent of patients who had a history of contact and/or clinical findings were hospitalized in YYU Hospital [7]. Tens of thousands of chickens were culled [8].

Avian influenza infections in Turkey provide an example of concurrent animal and human avian influenza epidemics. These were the first occurrences of human cases in the country, and the impact of the epidemic was quite strong. It was the main agenda for the local and national institutions of health, agriculture and other sectors, the community and the media during the first months of 2006.

This study aims to understand the course of the avian influenza outbreak control in Turkey through the views of health care providers. It will also highlight what local health personnel in various positions experienced during that period.

## Methods

This qualitative study was carried out in Dogubeyazit and Van in May 2006. We interviewed seventeen key informants who took active roles during the outbreak of avian influenza in East Turkey during the first months of 2006. The twelve of interviewees were medical doctors (directors, specialists and general practitioners), three were allied health personnel (one director and two health officers), and two were midwife and nurse (primary health care provider and director). Most of the informants were senior staff and had primary responsibilities for the management of the outbreak control.

Five informants were from the Van Provincial Health Directorate (PHD). These informants were the Director and the Deputy Director of the PHD, who were the main coordinators of the avian influenza outbreak intervention, two department chiefs who were involved in implementing the intervention and one health officer who is responsible for transportation of the samples and record keeping.

Four informants were health personnel in primary health care centers; three general practitioners and one midwife, who have active roles in surveillance in Van and Dogubeyazit. One of the general practitioners made the initial diagnosis of the avian case at a primary health care center in Van. Two general practitioners were staff of the primary health care center in Dogubeyazit. One of the doctors were the chief of the primary health care center and worked as a coordinator of avian influenza outbreak intervention and the other one was assigned to the avian influenza surveillance.

Three informants were staff of the State Hospitals in Van and Dogubeyazit. The first was an infectious disease specialist working in Van State Hospital. The second was the Director of Dogubeyazit State Hospital during the outbreak. The third was a pediatrician working in Dogubeyazit State Hospital who pre-diagnosed the avian cases in Dogubeyazit. They worked as if gate-keepers between the primary health care center and the YYU Hospital during the outbreak.

Five informants were Van YYU hospital staff. They were the Director of the University Hospital, the chief of nursing staff, two specialists and one health officer. The infectious disease specialist is on duty in the infectious disease control committee of YYU Hospital, who was a counselor in the Van Crisis Coordination Committee (CCC) and communicated with WHO representatives for the outbreak control. One internal medicine specialist who was also a native of Dogubeyazit helped the Provincial Health Directorates and WHO representatives to communicate with families from Dogubeyazit. The health officer was assigned to the emergency services during the outbreak and he was also the representative of the Health Workers Union in Van.

We used a semi structured guide consisting of open ended questions during in-depth interviews. All interviews were audio-taped and field notes were taken afterwards from each interview for data-gathering. The appointments with the informants were arranged by the second author of this study, who worked in Van Yuzuncu Yil University, and the interviews were conducted by the first author, who worked outside the region. The authors clarified the purposes of the study and the interviews were conducted with the informants' consent for audio-taping and for the use of their words in the report. In the institutions where the authors were affiliated, interview studies are not subject to permissions from ethical committees. Thus, the authors did not apply for approval of these institutions.

Following the interview guide, we asked the participants to inform us about the events chronologically: the first responses of the organizations, the management of outbreak control, the reactions of the health personnel and the resident population, the inter-organizational coordination, and interviewees' roles during the avian influenza outbreak in Turkey. We also asked them to evaluate the strength and weakness of the outbreak control, and the lessons from the avian influenza outbreak.

Records were transcribed verbatim. The authors read and identified codes for major themes according to their interest for the study. These codes were then marked to lines of text and the relevant codes were collected one under the other for preliminary analysis. The second analysis involved recovering the relevant codes from the text. The results of the study are reported according to the topics revealed by the analysis.

## Results

In the avian influenza outbreak, the studies by health organizations were defined by two features as suggested by the interviewees: it was perceived as a new and unknown disease; and it was encountered as a regional, national and international crisis by the health authorities. The preparedness of animal and human health organizations for outbreak- human resource, management, surveillance approach and notification systems- affected inter organizational coordination during the outbreak.

### Avian influenza infection as a new and unknown disease

Indeed, avian influenza infection was perceived as new and unknown disease by health care providers a consulted paediatrician from Dogubeyazit Government Hospital explained as: *" On December 31^st^, my colleague called and said that there were 'some patients in here who've been coming for three days now, and their conditions are very serious'. The chest x-ray was very bad, decreased leukocyte and thrombocyte counts in the laboratory analyses. I said it was possibly a viral disease (...); 'an epidemic, if four people have caught it. There is little that we can do here because of Dogubeyazit's limited facilities. We have to refer them to the University Hospital.' (...)"*.

The events that took place after the referral of suspected cases from Dogubeyazit to YYU Hospital were explained by the Institution's Director: *"I learned that some children had a viral infection that didn't match atypical pneumonia. I connected paediatricians and they said yes, 'it may be bird flu, SARS or something else (...) this is a significantly different disease that advances rapidly'. They said 'we're confused, there's not much we can do, we have no opportunity for detailed analysis, but we are still investigating (...)". We informed the governor, gendarme and security forces; they're checking entrances and exits" *Following this information, Van CCC decided for animal quarantine, and entrances and exits of poultry to the region were forbidden.

The general practitioner who pre-diagnosed the patient defined the perceptions of health care providers towards an unknown disease: *"Frankly, I did not believe in bird flu that much. I thought that this disease would never come to Turkey from its Far Eastern origins (...) that day my patients came, two sisters, the younger one was 8 and the elder was 25. They said something like, 'our chickens have started to die, and I wondered if it was the bird flu that we've seen on the TV'. I looked at the child and there was no clinical finding except conjunctivitis. Now, on the one hand, there was something that I don't believe, and on the other hand I had sisters talking about dead chickens (...) At that point, I checked my conscience. I said, all right then; let's consider these as bird flu cases. I gave the notice to the Van PHD immediately"*.

### The response of the avian influenza outbreak and associated problems

After laboratory confirmation of the suspected cases from Dogubeyazit, trace-back investigations initiated by the Local Health Authority were converted by the Turkish MoH intended zoning directed to the source of infection. Oriented by these instructions, doctors were assigned with the surveillance in Dogubeyazit from various parts of Turkey. The process was explained by a general practitioner who is responsible for the avian influenza surveillance from the primary health care center (PHCC) in Dogubeyazit: *"In the beginning, we thought that it would be best to visit the neighbourhoods and villages in which the cases were encountered and then visit the rest of the area in the remaining time. Just half of Dogubeyazit had been scanned in a period of 15 days. Later we were told that field studies would continue until the end of April and that a new health team would be sent, but nothing like that happened. Field studies were stopped when the number of new cases lessened."*

Health personnel supported to the field studies and well communicated with community during the surveillance in Dogubeyazit. However, the deployment of these studies and harsh weather caused some transportation problems. A general practitioner participating in the field studies in PHCC explained the efforts for these problems solving: *" The doctors worked very hard. Because it was snow, studies in villages could be made away slowly (...) We encountered serious basic logistic problems. Importantly, no extra budget was given to the primary health care centers for the field studies. The equipment was sent to the State Hospital by the MoH. We had a lot of vehicle trouble. We managed to compensate with vehicles or ambulances from the Dogubeyazit District Education Directorate, Governorship and Municipality. Things were better at the center."*

WHO team, experienced in influenza outbreaks in different countries came to the region during the outbreak. WHO experts investigated the animal to human transmission in the region and helped the Turkish authorities to develop the strategies for the outbreak control. A specialist working in the Van CCC as a representative from Van YYU expressed the importance of the situation through the evaluations of WHO representatives: *"There was well communication between the Health Directorate, WHO and YYU all the time. WHO teams thought that the surveillance system was not installed and patient records were not being kept in order. Indeed, a group of patients come to our hospital with certain complaints and we evaluate them. We hospitalize them if necessary, and there is another considerable risk group to whom we give prophylaxis. But we can't follow the group that receives prophylaxis. Nobody cares if something happens to them later."*

The comments related to follow up show us that there is an inconsistency between the pandemic influenza action plan and avian influenza surveillance approach. Although a circular of Turkish MoH was announced on October 2005 including a flow chart to evaluate the suspected cases, the exclusion of primary health care centers and hospitals in the sentinel surveillance and the lack of regional preparedness might have caused health care workers to miss some cases in the referral and reporting procedures.

The problems during the follow up studies were verified by the Health Directorate Deputy Director: *"The problem was this: the disease identified but surveillance couldn't have been initiated (...) At that time, the Turkish MoH didn't have a health record system for surveillance by the primary health care centers. So the practitioner needed to record cases on those forms so they would be reported to the second stage health care services."*

After the confirmation of the first patient by Ankara Hifzisihha Laboratory, a patient data form for surveillance was developed by the Van PHD communicable disease department and sent to the PHCCs of the region to follow up the suspected avian influenza cases. Later, however, the Turkish MoH sent new surveillance forms, but these were changed after WHO's proposals were adopted and the final guideline was then developed. The contribution of WHO team was helpful for the standardization of the form but the differentiation between the surveillance forms after from the avian influenza outbreak raised confusion as the Director of the Communicable Diseases Unit in the Van PHD stated: *"(...) the primary health care workers were also confused because the forms had been changed four times. There had been a lack of coordination concerning the surveillance forms. In my opinion, to maintain standardization, the Turkish MoH should have sent the forms much earlier"*.

After the confirmation of the suspected cases, Van Crisis Coordination Committee assembled committed an important role in managing the outbreak by well-timed decision making and by ensuring the participation of different organizations.

Because the outbreak threatened human and animal health, the coordination of studies by the Turkish MoA and Turkish MoH organizations became a vital issue. The lack of infrastructure and the experience of outbreak control emerged serious difficulties between health and agriculture organizations. Turkish MoA was not conducted the animal surveillance in accordance with the laboratory results of human cases. This issue was explained by the Van PHD Director: *"Normally, to start culling, the Van PAD announces that a site is infected. After that, health care providers conduct an operation within the site. But, the reverse has happened in here. First, we found our case and reported to the Van PAD. We said, 'Here is an infected site, you have to start the surveillance' and agriculture teams started culling the next day"*.

The Van PAD tried to overcome the personnel insufficiency by purchasing services. In the meantime, both the people and the agriculture providers have contamination risk in the infected region. A deputy director from the Van PHD who coordinated surveillance in Van stated that: *"We saw dead chickens in coops during the field study, doors open, and no disinfection. Everywhere was covered with snow and the coops had become a playground for children (...) MoA teams decided to purchase services and veterinarians joined in. Some cullers had no protective equipment. In most areas, hosts held their chicken and it started to struggle, its saliva was scattered around. There was high contamination risk"*.

Restricted surveillance proceeded in Dogubeyazit and were carried out uncomplicated with the reinforcement by the health care providers coming from outside the county. However, there was a different problem here. A health officer from YYU Hospital stated that the Turkish MoA teams did not conduct a standard operation and that this caused economic loss of the poultry owners:

*"People live on chicken and eggs in this region. Now, suddenly, all the chickens were sick and they were slaughtered in panic. No distinction was made as to whether a bird was healthy or sick. Economically, this shouldn't have been done to people living here. This may completely confuse people in this region, who hardly have anything left"*.

The cost of the culling animals was partially paid to the owners to compensate their economic loss. But in practice the official records couldn't be made exactly, therefore most of poultry owners could not receive the compensations and they had been subject to high rates of financial loss. The situation was expressed by the general practitioner from the primary health care center in Dogubeyazit:*"Now about 90–100 thousand chickens were slaughtered. Five Turkish liras were to be paid for each but only 30% of this price was paid because 70% of them were taken without any official record so people couldn't claim their rights."*

### First reactions of the health care providers and problems

After the declaration of avian influenza outbreak by the Turkish MoH, people's reactions to the disaster and patient applications increased to health care centers. The fear and panic were experienced among health care providers. This situation was explained by a health officer from YYU hospital: *"When cases arrived in Van we faced unbelievable panic and fear. There was no information related to the situation. Sometimes other staff was affected by clinical personnel wearing a mask while walking outside the hospital. Suspected avian cases were brought in the emergency room side by side with other patients. Now, health care providers were agitated and excited, not knowing what to do"*.

Initially, the health personnel's behaviour affected the people's psychological status. The statement by the infectious disease specialist from Van State Hospital was as follows: "*Health personnel were anxious, they panicked. Everybody wanted Tamiflu. No, I gave it to no one, including myself. They were convinced after the information gathering"*.

A health officer, who was getting the samples and transporting it to laboratory, expressed his anxiety and desperation due to the lack of protective equipment as follows: *There was no clear information (...). We couldn't protect ourselves at the beginning. We were used to the non-protective gloves and masks. Later, I wore two pairs of gloves. I know that it does not spread from human to human, but there is a fear that cannot be conquered"*.

The delay in the protective equipment support was the main reasons for the worries of the health personnel working in the wards: *"Patient aspiration, mouth care, intravenous, drawing of blood. All of this was done by our team and our health personnel grumbled because we didn't have protective glasses. After it was made known by the media, equipment was supplied to us, but it wasn't sufficient (...) We could have been informed earlier, in fact. This disease is contagious and deadly (...) We only had simple surgical masks. Despite that, we used gloves, coats and bonnets." *After the chaos of first day the Turkish MoH provided personal protective equipment for the health care providers in the region.

In Dogubeyazit, the main problem was the distribution of protective equipment, as well as providing it. A midwife who took part in the field studies stated her worries about contamination risk of her own family members and how this affected their efficiency at work: *"We went to the surveillance for suspected cases, but masks and glasses reached us after the work was finished (...) I was very sad when I heard that we would go to a suspected case. What if I get infected, if I transport it home? I have little kids. I think if they were given to us before, more efficient work could have been achieved, I guess."*

### The participation of the people during the avian influenza outbreak and trust in governmental authorities

The relatives of other patients were affected psychological because their children were treated in the same clinic by avian flu patients. They wanted to discharge from hospital. Their reactions, according to the chief of nursing staff, were as follows: *"Relatives of the patients were anxious. They tried to take their children we had transferred to other rooms. Everybody was anxious. The greatest anxiety concerned whether human-to-human transmission was possible."*

Relatives of avian influenza patients faced many difficulties because of communications with the health care providers in the hospital. The words of the general practitioner reflects us the human side of the disaster and the psychological status of the affected people. *"But what a trauma, a disaster, darkness. I enter a room and get goose pimples, a mother and her seven children. I take the patients' history, but she doesn't know Turkish; she doesn't say anything and then starts a Kurdish elegy, just like I've seen on TV. As if she's in a trance. The mother with her 7–8 kids and they don't know to whom the death will come. Just waiting like that, terrible psychology."*

Traditional family-based poultry farming in that region is the largest food and income resource. According to the director of the Van PHD at that time, people were initially unwilling to hand over their animals although declarations were alarming. After informative media operations, they helped to the culling operations. Crisis centers assembled at the Van PAD and PHD by the decision of the Van CCC were kept operational 24/7. National and local media organizations announced the phone numbers for people having questions of any kind: *"For instance, agriculture personnel had serious problems during the field study from time to time. Some of the people didn't want to hand over their chickens. 'My animal is healthy, why are you taking it?' they were generally saying (...) In this region there is no professional poultry farming. We informed them that today's healthy chickens may become ill tomorrow, and it threatens all the family, especially children. Of course, as a result of these informative operations people now denounce themselves"*.

The people's response to the operations of health and agriculture organizations and their participation is also related to their trust in governmental authorities. It is known that the municipality and local health care providers committed important functions in communicating with the public and in overcoming the obstacle of distrust: *"We hardly convinced him; we couldn't convince him about serosurvey operations for a month and called the municipality provider (...) Anyway, native people consider you a stranger but they know this region's people. They've helped us a lot; the municipality convinced him*.

Sometimes, people consulted to the non-governmental organizations: *"A farmer called me, called the health care provider union and found the phone number and asked 'should we slaughter our animals or not? Bury them? What should we do? We don't have any confidence left in authority.' He is confused. This made me think a lot, however I was very happy that a citizen trusts a non-governmental organization and is asking something."*

### Lessons from the avian influenza outbreak and suggestions by health care providers

The health care providers we have interviewed mentioned that human cases presented direct contact histories with domestic birds, which is part of the lifestyle here. Direct contact with diseased animals or waste from slaughtering is basic reason of contamination, especially for children. A director working in the Van PHD drew the sheltering status for the avian influenza patients: *"There was a close contact history for all those kids. The K. Brothers' home has only one room. At night, because it's winter time, they bring in the chickens in cardboard boxes, and look after them in there, sleeping together. A high contact risk is present."*

An infectious disease specialist defined the risk of contamination: *"Exposure to viral loads, the amount of infectious agent, is lower in older people because children deal with chickens, one-to- one. In most of the H5N1 positive cases, the chicken gets ill and when they understand that it is going to die, they decide to cook and eat it. Who's going to clean it and cook it? The youngest daughter, of course. Big brother slaughters it and leaves it like that. Younger sister cleans the animal."*

During visits to the house of the avian influenza patient, the general practitioner from the primary health care center evaluated the habitat such as the following were: *"I observed this at the front of S.'s house. Why have all members of the family caught influenza? There is a small river, a bad one, which passes behind their house. There is nothing like a sewerage system or toilet, it is directly connected to the river. There is no clean water in the house. That day S. had hugged the chicken but her uncle's daughter had picked it. She couldn't have washed her hands properly. There is a very serious hygiene and infrastructure problem."*

Health status, sheltering, infrastructure problems of community were related with H5N1 transmission in Dogubeyazit and Van, as explained by a general practitioner: *"I've observed here, the reason of the deaths is not the avian influenza virus; it is poverty, chronic diseases, and negligence of children. Most dead were children, and some didn't even have ID's (...) Chickens were slaughtered on open ground. Those dead children played with the chickens, they played with their heads as puppets, and they slept together in the evening."*

According to the health care providers' experiences, to deal with an outbreak, the health system must be strengthened, health services should be coordinated, surveillance system and the notification for communicable diseases must be operated efficiently, human and animal health care should be integrated.

Health personnel suggested that the preparedness of the health organizations is very important for an outbreak in terms of equipment, human resources and information gathering The preparation studies should not only be on the management level, but also throughout the health organization and the experience of public health workers must be taken into account:

"In such a disaster you should be ready with your team. Even if the human resource are not enough, teams would be ready, patient data forms should be available immediately, surveillance and a health team, and, of course, communication with the ministry, to make sure that the supplies are brought to the place."

"Field studies are very important. The opinions of public health workers are much more valuable than what the health director said (...). I mean, PHDs should take care of public health workers envision."

*"The 1961 Act on the Socialization of Health Services states that a midwife can take care of a population of up to 2000–2500 people. At Van, there is still one nurse or midwife caring for 7000 people. This is the case for the primary health care services currently. (...) Our health record and notification system is not fully installed yet"*.

"A little bit of knowledge and understanding of rural areas and rural life is needed. People who came from urban areas are not aware of the picture. Who will work where, how the organization will be established and to command of the health personnel are very important."

Some of the suggestions of the health care providers about surveillance were:

*"If you follow up an outpatient, you have to visit them every day. It cannot be done by the state or the university hospital. This must be reported to primary health care centers*.

*"My opinion is that continuing education of health care providers should be provided, so the practitioner who notices a suspected case can take the details of the patient's history. If it is done like this, the system will work better, a recording system is formed"*.

According to the experiences of health personnel, sectors and institutions related to the outbreak control must work in collaboration and be coordinated: *"(...) avian influenza control absolutely a multi-sector activity. No one should try to be a hero. Every health organization continues with its task; if the organizations of agriculture, municipality, gendarme, police, mufti, education are not included, your success will be overshadowed, and your aim is not achieved."*

The subjects that most stressed by the health personnel were the prevention of transmission from animal to animal and from animal to human. The health personnel agreed that besides culling animals, studies on preserving animal health must be developed and sustained. In place of traditional poultry farming, alternatives suggested included changes to modern poultry farming at nearby settlements:

"At this time our advice to the Van PAD was that since all of them were culling, new projects must be developed and information should be gathered on poultry farming. Of course, this should be done with government support."

"The origin oriented control should be. Firstly, a surveillance system at our borders must work well, especially in terms of animal surveillance, but also mainly before disaster happens, not only when a pandemic or epidemic occurs (...)."

As the health personnel, communication and information gathering is very important for the outbreak control. The public's psychology and needs must be taken into account to enable them to cooperate with the health teams. The general practitioner who has a close relationship with the local people emphasized the importance of people's psychology and environmental factors. *"I mean, why wasn't 'a child playing with chicken heads like puppets' put on the agenda? When she was discharged from the hospital, she came to visit me and I bought her a doll and advised her not to play with chickens, which are microbial things. She slept with the doll for many nights. Why didn't anyone underline this occasion? Why was the people's psychological status not taken into account? Why wasn't attention paid to the problems related to the housing infrastructure or the lack of clean water? I still can't accept this; it still affects me."*

## Discussion

The Turkish Avian Influenza outbreak is an example for other countries in respect of the experiences and lessons achieved by the health organization managers and health care providers who actively worked in the process.

The health care providers who participated in our study think that the avian outbreak reached national and regional crisis level because they faced an unknown emerging infectious disease and the threat to health that it was not predicted.

According to the health care providers, the most critical issue was the coordination between the agriculture and health organizations during crisis control. The main reason for this problem was the lack of organizational preparation compatible with central or regional plans. To be prepared for an outbreak, it is vital to define the central, regional and local organizational framework, to maintain close cooperation and collaboration between the health and agriculture sectors and to share information on surveillance, evaluating the risk to humans and planning interventions at the time when a case occurs [9-12]. WHO indicated that Turkey's preparedness plan provided a framework for action, even if not yet fully developed [3].

Indeed the Turkish MoH published the country's first national pandemic influenza action plan in October 2005 and a circular was sent to health organizations about possible avian influenza case descriptions and precautions [3,8]. On the other hand, the results of this study indicates that, the circulation of the avian influenza action plan was insufficient, and regional and national activities related the outbreak control only started after following the verification of H5N1 virus in poultry. As preventive human and animal health care, organizational preparations were incomplete. WHO states that there was no active surveillance in neighbouring provinces after a domestic animal outbreak was confirmed in Igdir in December 2005 [3].

According to the health care providers who participated in our study, a reference laboratory could not meet the demand for the animal samples and after the human cases arose, the Turkish MoA decided to cull all the poultry without searching for possible or confirmed cases. Because there were not enough agriculture personnel for the culling at Van, the Turkish MoA purchased support from private veterinary clinics. This shows us the size of crisis and the lack of preparedness such as reviewing organizational facilities and operational plans in pre-outbreak phase. As a result, the work of outer-organizational teams, which proceeded in their own region, was not integrated with that of other regional health teams that continued surveillance. The evaluation of animal health services at that period is limited in this study because we did not interview agriculture personnel. Future research should focus on the experiences of the animal health service personnel.

Evidently, chaos is unavoidable in primary health care services that do not include central and organizational level intervention plans for emerging avian influenza-like infectious diseases, as in the Turkey example. The health care providers think that the strengths and weaknesses of the health organizational structure before the outbreak affected the success of the intervention and problems were encountered in coordination between health institutions. While a failure is expected even in the health services of developed countries following a possible avian influenza pandemic [13,14], the chaos can be bigger in developing countries due to the weaknesses of health organization. Therefore, strengthening the healthsystem in developing countries shouldbe considered as a factor in delaying the worldwide spread of avian influenza. National and international network and partnerships have to maintain current public health and animal health infrastructures and resources for construction, modernization, enhancement and recruitment for unprecedented emerging and re-emerging disease [15].

The most critical issue related to the health system is the surveillance system. The notification of communicable diseases in Turkey was changed in recent years. In the new system, influenza group diseases are subject to sentinel surveillance. According to the sentinel surveillance, primary health care centers have no obligation to notify influenza group diseases including the possible avian influenza cases. Only the training and research hospitals in some selected provinces have the duty for notification of confirmed influenza cases. During the Turkish avian influenza outbreak, Van and Dogubeyazit (Agri) were not included among the selected provinces.

The health care providers who participated in our study emphasized that the widespread of the outbreak requires follow-up and assessment of all influenza cases who applied to health care centers. As the interviewees stated, trace-back investigations were not performed because of the current surveillance system mentioned above. The need for an active surveillance system is clear for an epidemic disease such as avian influenza, which is seasonal, regional and closely related to epidemic diseases related to agricultural practice. In the WHO Report dealing with lessons from the avian influenza outbreak in Turkey, the need for active surveillance of animal and human avian influenza outbreaks was underlined [3]. A well integrated effort such as lining up a network between animal and public health laboratory system is needed to define an epidemic earlier, ensure more effective control measures [7,15]. This will possibly make us gain valuable time by delaying a pandemic.

Practitioners, midwives and nurses, who are responsible for population-based health care services in the primary health care system, became the most important human resource for surveillance during the avian influenza outbreak in Turkey. This experience clearly shows that primary health care centers should be included in the surveillance system. For a efficient surveillance system, upgrading the sentinel physician network by enlisting and retraining more participants and [16] the coordination among public health workers, clinicians and managers is most necessary. It is accepted that public health workers will play an integral role in an influenza pandemic [17]. The health managers and providers whom we interviewed believe that their experiences must be reflected in national avian influenza preparedness plans.

Another problem encountered during the avian influenza outbreak in Turkey was the fear of contamination risk among the health care providers who have no protective equipment against H5N1 when the outbreak started. It is as important to train the health personnel to be prepared for an outbreak as it is to stock protective equipment for them at the sites of preparation studies [14,15,18]. Interviewees believe that the delay in supplying protective equipment and the lack of orderly planning in the distribution of it caused concerns among health care providers about contamination risk, which affected their work output. Health personnel must be accepted as the primary group in prevention, and mortality and morbidity among them must be minimized to allow for efficient intervention by the maximum number of personnel during an avian influenza pandemic. Even if epidemiological evidence about the spread of the H5N1 virus from patients to health personnel is limited [19], studies indicate that nurses who work in hospitals and public health workers touching the suspected avian influenza cases may experience fear and anxiety for their own and their families' health and can face ethical dilemmas when deciding between continuing their work and maintaining their families' health [20,21].

Several problems were experienced during intervention in the outbreak by health care providers who participated in the study. They may be classified as problems related to the socio-economic conditions of people living in the region, and those related to the health care system, including the management and surveillance systems:

The health care providers emphasized that people who lived in the rural part of Dogubeyazit and the suburban areas of Van that gather migrants lacked basic needs such as health, education and infrastructure. According to the interviewees, the outbreak in Turkey was influenced by factors closely related to traditional poultry farming and poverty-line economics, as was the case in Asian countries [22]. According to the health personnel who participated in our study, the infection spread rapidly because domestic birds belonging to neighbourhood backyards were able to walk around freely. It is possible that wild migrant birds may be in contact with poultry in the area for food or water. Family-based small-scale poultry farming is a major source of income and nutrition for the region.

All our interviewees thought that the most important factor in the transmission of the H5N1 virus from animal to human was the sharing of shelter according to the low socioeconomic status of the family. Especially during fall and winter, family members share their one sleeping and dining room with poultry. From the year 2004, the contact histories of children and young adults who are described as a risk group for avian influenza cases throughout the world resemble the ones in Turkey [23]. Health care providers who worked in Van and Dogubeyazit stated that people had eaten ill chickens before death and young adults had taken part in the cleaning, cooking and slaughtering of the chickens. Children had played with sick animals and corpses.

The low socio-economic status of the people was accompanied by insufficient primary health care services in the region. The first avian influenza cases were diagnosed after lower respiratory tract infection symptoms appeared. All the fatalities were among those who had recently applied to a health center and been diagnosed as avian influenza. Informants have differing explanations for the late diagnosis of the patients. According to some, the first admission of the families was late. Others claim that the family of the first cases was sent back home during the first admission, and avian flu was diagnosed only after their second admission.

The community was affected psychologically and economically from the disaster. Avian influenza created the fear of a mysterious disease on the people. Most of the people were subject to a financial loss because of the slaughtering of their poultries and were impoverished relative to previous status. In this study we found that the communication between community and health personnel is important for establishing public support for outbreak control and to overcome obstacles such as lack of confidence in governmental organizations. After the avian cases were verified in Turkey, the declaration by the Turkish MoH was evaluated affirmative by the health care providers. It is very important that managers be clear about the known and unknown issues by the first stage of the avian influenza crisis, inform the people about health risks and precautions as early as possible, and be sensitive the worries of people [24,25].

According to the health personnel interviewed, the reason why poultry were kept as a supply of food and living in Van and Dogubeyazit at the time the culling started was the lack of public education about preventable health problems in the region, as much as economic security. The experiences of health personnel indicate that the people participated more after the crisis centers were established by the health and agriculture boards, phone counselling services were made available, good communication was established between health personnel and the people during surveillance, and information studies were applied by media channels.

## Conclusion

Turkey experience in avian influenza outbreak shows that preparation planning and surveillance systems should be rational and sustainable. The lack of organizational emergency disease plans delineating the tasks and responsibilities of health care providers in the event of a possible avian influenza outbreak, and the lack of training about preventive care, caused health personnel to be caught unprepared by the outbreak. The problems in supplying and distributing protective equipment, reflecting another dimension of the lack of organizational preparedness, influenced the efficiency of the health care providers' work because they were anxious about their own and their families' health. During the preparation and updating of national epidemic and pandemic plans, and during outbreak management, public health workers should participate effectively in decision-making and risk communication. A well designed communication strategy people's participation in control and may relieve from the psychological effects of the outbreak.

Animal and human health care services could not be well coordinated owing to the lack of integrated preparation and planning. Because the sentinel surveillance of influenza infections in Turkey was based on the confirmation of the cases diagnosed at the training and research hospitals in selected provinces, detection of possible human avian influenza cases at the primary health care level was hindered and trace-back investigation was precluded. To evaluate the spread of the avian influenza outbreak accurately, evaluation and follow-up of influenza cases needs to be done in all health care centers. Such evaluation also necessitates integrated human and animal surveillance and control measures.

The causes of transmission of the H5N1 virus from wild to domestic animals in Turkey are closely related to socioeconomic conditions and traditional poultry farming. Poverty in the region played a predisposing role in the outbreak and the outbreak also increased poverty. Limited access to education, shelter, water supply and waste removal formed the basis for virus transmission from animals to animals and animals to humans. To reduce the risks rising from these predisposing conditions, infrastructure needs to be built specially at the village level.

Although the limited access to health care services and the high contact rate with ill chickens were among the characteristics of the vulnerable group, the rapid organization of the health authorities during intervention studies prevented an increase in the mortality rate. Despite the above-mentioned problems in preparedness about coordination during the avian influenza outbreak control, the rapid response and performance of the health workers played an important role in controlling epidemic.

## Competing interests

The author(s) declare that they have no competing interests.

## Authors' contributions

Both authors contributed to the design of the study. OS worked on data gathering and data analysis and drafted the manuscript. TE arranged appointments with the informants during the course of data gathering and contributed to analyzing the data. Both authors read drafts of the manuscript, made comments and approved the final version.

## Pre-publication history

The pre-publication history for this paper can be accessed here:


